# The effects of β-glucan on human immune and cancer cells

**DOI:** 10.1186/1756-8722-2-25

**Published:** 2009-06-10

**Authors:** Godfrey Chi-Fung Chan, Wing Keung Chan, Daniel Man-Yuen Sze

**Affiliations:** 1 Department of Paediatrics & Adolescent Medicine, Li Ka Shing Faculty of Medicine, The University of Hong Kong, Hong Kong; 2 Department of Health Technology and Informatics, The Hong Kong Polytechnic University, Hong Kong

## Abstract

Non-prescriptional use of medicinal herbs among cancer patients is common around the world. The alleged anti-cancer effects of most herbal extracts are mainly based on studies derived from *in vitro *or *in vivo *animal experiments. The current information suggests that these herbal extracts exert their biological effect either through cytotoxic or immunomodulatory mechanisms. One of the active compounds responsible for the immune effects of herbal products is in the form of complex polysaccharides known as β-glucans. β-glucans are ubiquitously found in both bacterial or fungal cell walls and have been implicated in the initiation of anti-microbial immune response. Based on *in vitro *studies, β-glucans act on several immune receptors including Dectin-1, complement receptor (CR3) and TLR-2/6 and trigger a group of immune cells including macrophages, neutrophils, monocytes, natural killer cells and dendritic cells. As a consequence, both innate and adaptive response can be modulated by β-glucans and they can also enhance opsonic and non-opsonic phagocytosis. In animal studies, after oral administration, the specific backbone 1→3 linear β-glycosidic chain of β-glucans cannot be digested. Most β-glucans enter the proximal small intestine and some are captured by the macrophages. They are internalized and fragmented within the cells, then transported by the macrophages to the marrow and endothelial reticular system. The small β-glucans fragments are eventually released by the macrophages and taken up by other immune cells leading to various immune responses. However, β-glucans of different sizes and branching patterns may have significantly variable immune potency. Careful selection of appropriate β-glucans is essential if we wish to investigate the effects of β-glucans clinically. So far, no good quality clinical trial data is available on assessing the effectiveness of purified β-glucans among cancer patients. Future effort should direct at performing well-designed clinical trials to verify the actual clinical efficacy of β-glucans or β-glucans containing compounds.

## Introduction

A significant proportion of cancer patients have been taking complementary medical therapies while receiving their conventional anti-cancer treatments [1-6]. Among them, herbal extracts such as *Ganoderma lucidum *are one of the most common modalities being consumed especially among Oriental [7-10]. Two mechanisms have been proposed to be responsible for the anti-cancer action of these herbal extracts; one is via direct cytotoxic effect and the other is indirectly through immunomodulatory action [11,12]. Many cytotoxic chemotherapeutic agents currently in use such as vincristine, taxol and etoposide are originally purified from herbs. On the other hand, herbs with immunomodulatory functions have mainly been advocated by commercial sectors and most of them can be directly purchased over the counter or the internet. Unfortunately, organized efforts to investigate the actual usefulness of this group of herbs as well as their active ingredients are lacking. In recent years, one of the active ingredients responsible for the immunomodulation of many of these herbs was found to be a form of complex polysaccharides known as "β-D-glucan", or simply called β-glucan [8,13]. The receptors and mechanisms of action of β-glucans have recently been unfolded via *in vitro *and *in vivo *animal experiments. Since β-glucans are inexpensive and have good margin of safety based on historical track records, their potential therapeutic value deserve further investigation. We reviewed here the literature and our experience on the *in vitro *and *in vivo *animal biological studies of β-glucans, particularly on their immune and anti-cancer mechanisms.

### Physical and chemical properties of β-glucan

**Figure 1:**
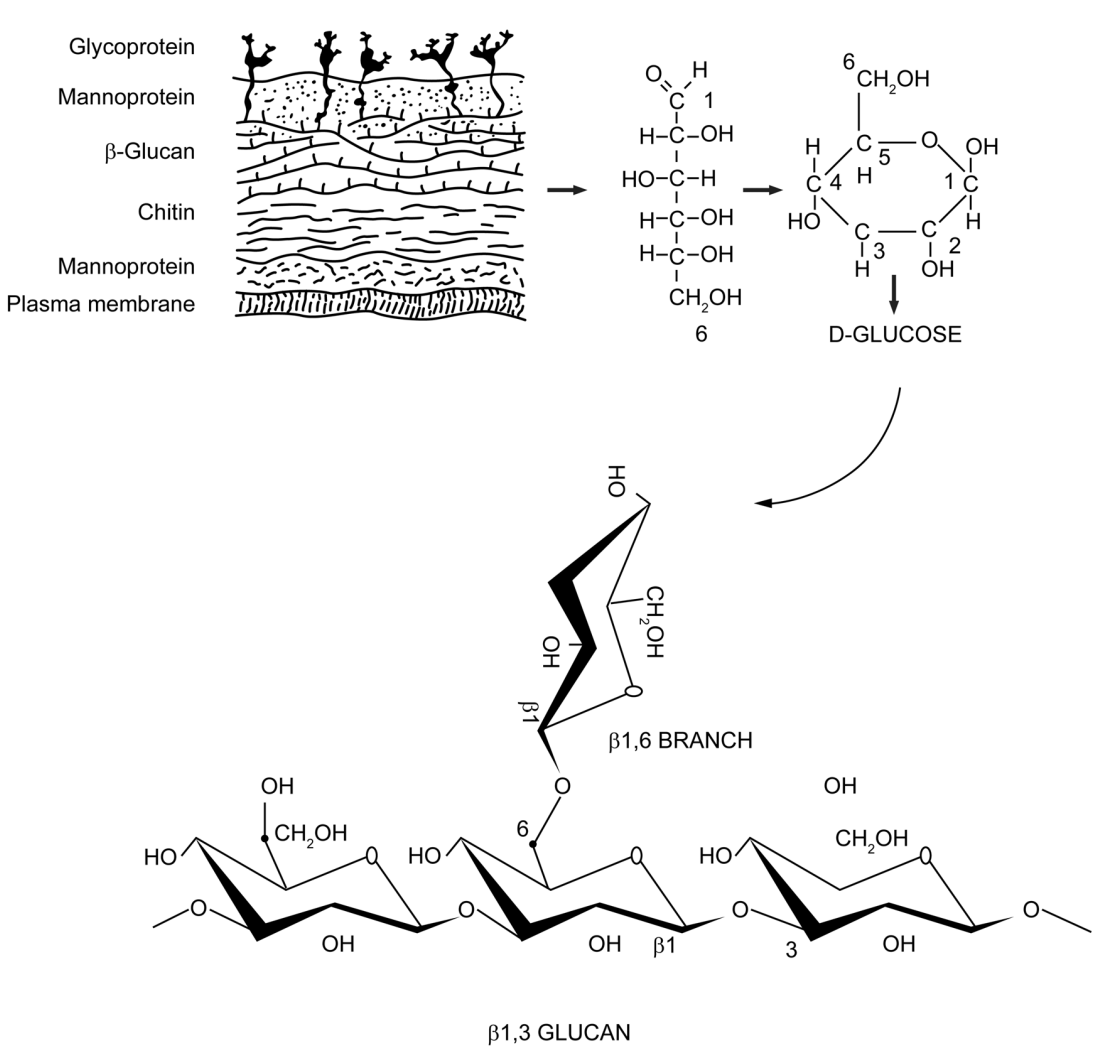
**β-glucan is one of the key components of the fungal cell wall**. The basic subunit of the fungal β-glucan is β-D-glucose linked to one another by 1→3 glycosidic chain with 1→6 glycosidic branches. The length and branches of the β-glucan from various fungi are widely different.

β-glucans are one of the most abundant forms of polysaccharides found inside the cell wall of bacteria and fungus. All β-glucans are glucose polymers linked together by a 1→ 3 linear β-glycosidic chain core and they differ from each other by their length and branching structures [14] (Figure 1). The branches derived from the glycosidic chain core are highly variable and the 2 main groups of branching are 1→4 or 1→6 glycosidic chains. These branching assignments appear to be species specific, for example, β-glucans of fungus have 1→6 side branches whereas those of bacteria have 1→4 side branches. The alignments of branching follow a particular ratio and branches can arise from branches (secondary branches). In aqueous solution, β-glucans undergo conformational change into triple helix, single helix or random coils. The immune functions of β-glucans are apparently dependent on their conformational complexity [15]. It has been suggested that higher degree of structural complexity is associated with more potent immunmodulatory and anti-cancer effects.

For research purposes, the composition or structural information of β-glucans can be evaluated by a variety of methods including liquid chromatography/mass spectrometry (LC/MS)[16], high performance liquid chromatography (HPLC)[17] and less often X-ray crystallography [18] or atomic force microscopy [19]. However, due to the tedious and lack of quantitative nature of most of these technical methods, they cannot be applied routinely as a screening tool. Other less sophisticated techniques in studying the β-glucans contents include phenol-sulphuric acid carbohydrate assay, aniline blue staining method and ELISA. Because chemical modification invariably induces changes in the natural conformation, most of these methods cannot reflect the genuine relationship between the structure and the bioactivity. Among them, aniline blue staining method is a relatively simple method to screen for β-glucan because of its ability to retain the natural conformation of β-glucans during the staining process. It also has a good specificity for β-glucans but its limitation is that it can only measure the core 1→3 linear glycosidic chain and not the branches.

Endotoxin contamination is another important issue affecting the safety and potential biological effect of β-glucan. Lipopolysaccharide (LPS) is an endotoxin found inside the Gram negative bacterial cell wall and consists of three main parts including lipid A, core and polysaccharide chain [20]. Among them, lipid A was found to be the major component that can initiate an immune response. LPS contamination can occur during the culture or preparation of β-glucans. Since LPS is one of the most potent immune stimulator and its contamination can lead to false positive results in immune tests, quantification of LPS should be performed, which can be evaluated by either the rabbit pyrogen test or the modified limulus amebocyte lysate (LAL) assay with devoid factor G [21].

### Pharmacodynamics & Pharmacokinetics of β-glucan

Most β-glucans are considered as non-digestible carbohydrates and are fermented to various degrees by the intestinal microbial flora [22-24]. Therefore, it has been speculated that their immunomodulatory properties may be partly attributed to a microbial dependent effect. However, β-glucans in fact can directly bind to specific receptors of immune cells, suggesting a microbial independent immunomodulatory effect [25]. The pharmacodynamics and pharmacokinetics of β-glucans have been studied in animal and human models.

#### Animal Studies

Study using a suckling rat model for evaluation of the absorption and tissues distribution of enterally administered radioactive labeled β-glucan, it was found that the majority of β-glucan was detected in the stomach and duodenum 5 minutes after the administration [26]. This amount rapidly decreased during first 30 minutes. A significant amount of β-glucan entered the proximal intestine shortly after ingestion. Its transit through the proximal intestine decreased with time with a simultaneous increase in the ileum. Despite low systemic blood levels (less than 0.5%), significant systemic immunomodulating effects in terms of humoral and cellular immune responses were demonstrated.

The pharmacokinetics following intravenous administration of 3 different highly purified and previously characterized β-glucans were studied using carbohydrates covalently labeled with a fluorophore on the reducing terminus. The variations in molecular size, branching frequency and solution conformation were shown to have an impact on the elimination half-life, volume of distribution and clearance [27].

**Figure 2:**
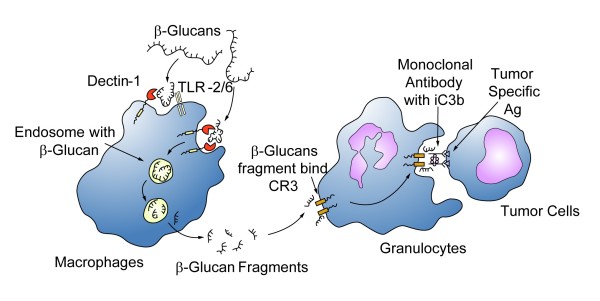
**The uptake and subsequent actions of β-glucan on immune cells**. β-glucans are captured by the macrophages via the Dectin-1 receptor with or without TLR-2/6. The large β-glucan molecules are then internalized and fragmented into smaller sized β-glucan fragments within the macrophages. They are carried to the marrow and endothelial reticular system and subsequently released. These small β-glucan fragments are eventually taken up by the circulating granulocytes, monocytes or macrophages via the complement receptor (CR)-3. The immune response will then be turned on, one of the actions is the phagocytosis of the monoclonal antibody tagged tumor cells.

The low systemic blood level of β-glucans after ingestion does not reflect the full picture of the pharmacodynamics of β-glucans and does not rule out its *in vivo *effects. Cheung-VKN *et al*. labeled β-glucans with fluorescein to track their oral uptake and processing *in vivo*. The orally administered β-glucans were taken up by macrophages via the Dectin-1 receptor and was subsequently transported to the spleen, lymph nodes, and bone marrow. Within the bone marrow, the macrophages degraded the large β-1,3-glucans into smaller soluble β-1,3-glucan fragments. These fragments were subsequently taken up via the complement receptor 3 (CR3) of marginated granulocytes. These granulocytes with CR3-bound β-glucan-fluorescein were shown to kill inactivated complement 3b (iC3b)-opsonized tumor cells after they were recruited to a site of complement activation such as tumor cells coated with monoclonal antibody [28] (Figure 2). It was also shown that intravenous administered soluble β-glucans can be delivered directly to the CR3 on circulating granulocytes.

Furthermore, Rice PJ et al. showed that soluble β-glucans such as laminarin and scleroglucan can be directly bound and internalized by intestinal epithelial cells and gut associated lymphoid tissue (GALT) cells [29]. Unlike macrophage, the internalization of soluble β-glucan by intestinal epithelial cells is not Dectin-1 dependent. However, the Dectin-1 and TLR-2 are accountable for uptake of soluble β-glucan by GALT cells. Another significant finding of this study is that the absorbed β-glucans can increase the resistance of mice to bacterial infection challenge.

#### Human Studies

How β-glucans mediate their effects after ingestion in human remained to be defined. In a phase I study for the assessment of safety and tolerability of a soluble form oral β-glucans [30]. β-glucans of different doses (100 mg/day, 200 mg/day or 400 mg/day) were given respectively for 4 consecutive days. No drug-related adverse events were observed. Repeated measurements of β-glucans in serum, however, revealed no systemic absorption of the agent following the oral administration. Nonetheless, the immunoglobulin A concentration in saliva increased significantly for the 400 mg/day arm, suggesting a systemic immune effect has been elicited. One limitation of this study is the low sensitivity of serum β-glucans determination.

In summary, based on mostly animal data, β-glucans enter the proximal small intestine rapidly and are captured by the macrophages after oral administration. The β-glucans are then internalized and fragmented into smaller sized β-glucans and are carried to the marrow and endothelial reticular system. The small β-glucans fragments are then released by the macrophages and taken up by the circulating granulocytes, monocytes and dendritic cells. The immune response will then be elicited. However, we should interpret this information with caution as most of the proposed mechanisms are based on *in vitro *and *in vivo *animal studies. Indeed, there is little to no evidence for these hypothesized mechanisms of action and pharmacokinetics occurred in human subjects at the moment.

### β-glucans as immunomodulating agent

**Figure 3:**
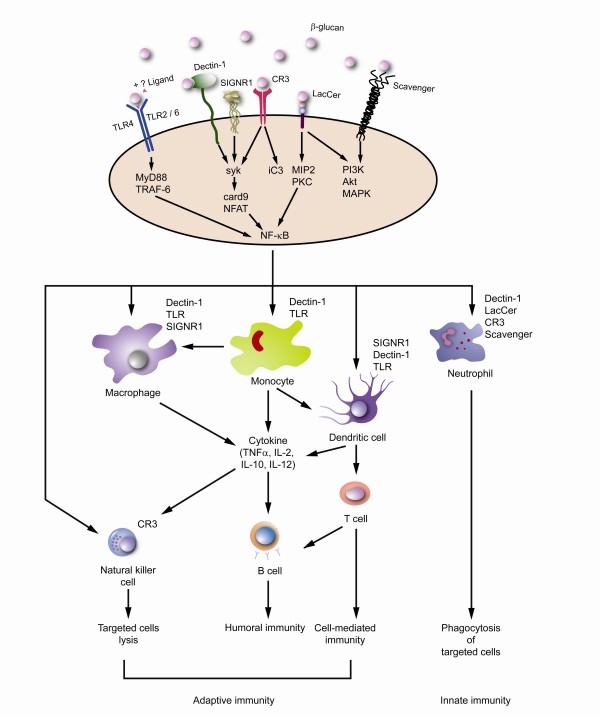
**Immune activation induced by β-glucans**. β-glucans can act on a variety of membrane receptors found on the immune cells. It may act singly or in combine with other ligands. Various signaling pathway are activated and their respective simplified downstream signaling molecules are shown. The reactors cells include monocytes, macrophages, dendritic cells, natural killer cells and neutrophils. Their corresponding surface receptors are listed. The immunomodulatory functions induced by β-glucans involve both innate and adaptive immune response. β-glucans also enhance opsonic and non-opsonic phagocytosis and trigger a cascade of cytokines release, such as tumor necrosis factor(TNF)-α and various types of interleukins (ILs).

Current data suggests that β-glucans are potent immunomodulators with effects on both innate and adaptive immunity. The ability of the innate immune system to quickly recognize and respond to an invading pathogen is essential for controlling infection. Dectin-1, which is a type II transmembrane protein receptor that binds β-1,3 and β-1,6 glucans, can initiate and regulate the innate immune response [31-33]. It recognizes β-glucans found in the bacterial or fungal cell wall with the advantage that β-glucans are absent in human cells. It then triggers effective immune responses including phagocytosis and proinflammatory factors production, leading to the elimination of infectious agents [34,35]. Dectin-1 is expressed on cells responsible for innate immune response and has been found in macrophages, neutrophils, and dendritic cells [36]. The Dectin-1 cytoplasmic tail contains an immunoreceptor tyrosine based activation motif (ITAM) that signals through the tyrosine kinase in collaboration with Toll-like receptors 2 and 6 (TLR-2/6) [34,37,38]. The entire signaling pathway downstream to dectin-1 activation has not yet been fully mapped out but several signaling molecules have been reported to be involved. They are NF-κB (through Syk-mediate pathway), signaling adaptor protein CARD9 and nuclear factor of activated T cells (NFAT) [39-41] (Fig. 3). This will eventually lead to the release of cytokines including interleukin (IL)-12, IL-6, tumor necrosis factor (TNF)-α, and IL-10. Some of these cytokines may play important role in the cancer therapy. On the other hand, the dendritic cell-specific ICAM-3-grabbing non-integrin homolog, SIGN-related 1 (SIGNR1) is another major mannose receptor on macrophages that cooperates with the Dectin-1 in non-opsonic recognition of β-glucans for phagocytosis [42] (Fig 3). Furthermore, it was found that blocking of TLR-4 can inhibit the production of IL-12 p40 and IL-10 induced by purified Ganoderma glucans (PS-G), suggesting a vital role of TLR-4 signaling in glucan induced dendritic cells maturation. Such effect is also operated via the augmentation of the IκB kinase, NF-κB activity and MAPK phosphorylation [43]. One additional point to note is that those studies implied the interaction between β-glucans and TLR all used non-purified β-glucans, therefore the actual involvement of pure β-glucans and TLR remains to be proven.

Other possible receptors and signaling pathways induced by β-glucans are less definite at the moment. For example, lentinan, a form of mushroom derived β-glucans, has been found to bind to scavenger receptor found on the surface of myeloid cells and triggers phosphatidylinositol-3 kinase (PI3K), Akt kinase and p38 mitogen-activated protein kinase (MAPK) signaling pathway [44](Fig. 3). But no specific β-glucans scavenger receptor has been identified so far. *Candida albicans *derived β-glucans but not other forms of pathogenic fungal β-glucans can bind to LacCer receptor and activate the PI-3K pathway in controlling the neutrophil migration [45] (Fig. 3), but such activation pathway may involve other molecules found in the Candida derived β-glucans.

We found that β-glucans can induce human peripheral blood mononuclear cells proliferation [46]. It can also enhance phenotypic and functional maturation of monocyte derived dendritic cells with significant IL-12 and IL-10 production. Similar findings were found by Lin et al. using PS-G, in addition, treatment of dendritic cells with PS-G resulted in enhanced T cell-stimulatory capacity and increased T cell secretion of interferon-γ and IL-10 [43,47]. This action is at least mediated in part through the Dectin-1 receptor. The potency of such immunomodulating effects differs among β-glucans and purified polysaccharides of different size and branching complexity. In general, bigger size and more complex β-glucans such as those derived from *Ganoderma lucidum *have higher immunomodulating potency.

The adaptive immune system functions through the combined action of antigen-presenting cells and T cells. Specifically, class I major histocompatibility complex (MHC-I) antigen presentation to CD8(+) cytotoxic T cells is limited to proteosome-generated peptides from intracellular pathogens. On the other hand, the class II MHC (MHC-II) endocytic pathway presents only proteolytic peptides from extracellular pathogens to CD4(+) T helper cells. Carbohydrates have been previously thought to stimulate immune responses independently of T cells [48]. However, zwitterionic polysaccharides (polysaccharides that carry both positive and negative charges) such as β-glucans can activate CD4(+) T cells through the MHC-II endocytic pathway [49]. β-glucans are processed to low molecular weight carbohydrates by a nitric oxide-mediated mechanism. These carbohydrates will then bind to MHC-II inside antigen-presenting cells such as dendritic cells for presentation to T helper cells. Initial data suggested that it subsequently leads to Th-1 response, but there are conflicting data related to this aspect. In our *in vitro *data, β-glucans do not tend to polarize T cells into either Th-1, Th-2 or regulatory T cells [46]. However, recent publications suggested β-glucans such as zymosan may induce T-cells into T-reg cells in a NOD mice model [50]. Therefore, whether β-glucans can induce important immunologic responses through T cell activation remain to be further investigated.

Another mechanism of β-glucan action is mediated via the activated complement receptor 3 (CR3, also known as CD11b/CD18), which is found on natural killer (NK) cells, neutrophils, and lymphocytes. This pathway is responsible for opsonic recognition of β-glucans leading to phagocytosis and reactor cells lysis. β-glucans bind to the lectin domain of CR3 and prime it for binding to inactivated complement 3b (iC3b) on the surface of reactor cells. The reactor cells can be of any cell type including cancer cells tagged with monoclonal antibody and coated with iC3b. The β-glucans-activated circulating cells such as the CR3 containing neutrophils will then trigger cell lysis on iC3b-coated tumor cells [28]. Similarly, majority of the human NK cells express CR3 and it was shown that opsonization of NK cells coated with iC3b leads to an increase in the lysis of the target. The beta chain of the CR3 molecule (CD18) rather than the alpha chain (CD11b) is responsible to the β-glucan binding [51,52].

This concept was supported by *in vivo *study demonstrating barley β-1,3;1,4-glucan given orally can potentiate the activity of an antitumor monoclonal antibody (anti-ganglioside-2 or "3F8"), leading to enhanced tumor regression and survival on a human neuroblastoma xenografts mouse model [53]. 3F8 plus β-glucan was shown to produce near-complete tumor regression or disease stabilization whereas 3F8 or β-glucan alone showed no significant effect. The median survival of the 3F8 plus β-glucan group was 5.5-fold higher than that of the control groups, and up to 47% of the mice remained progression free in contrast to <3% of controls at the end of the study period. No toxicities were noted in all mice treated with β-glucan, 3F8, or 3F8 plus β-glucan.

A similar xenograft model was adopted subsequently for investigating various targeted tumor antigens and tumor types. It was found that β-glucan exerts similar anti-tumor effects irrespective of antigens (GD2, GD3, CD20, epidermal growth factor-receptor, and HER-2) or human tumor types (neuroblastoma, melanoma, lymphoma, epidermoid carcinoma, and breast carcinoma) or tumor sites (subcutaneous versus systemic). The effect was correlated with the molecular size of the β-1,3;1,4-glucan [53,54].

Furthermore, 2 other receptors known as scavenger [55] and lactosylceramide [56,57] also bind β-glucans and can elicit a range of responses. β-glucans can enhance endotoxin clearance via scavenger receptors by decreasing TNF production leading to improved survival in rats subjected to *Escherichia coli *sepsis [58]. On the other hand, β-glucans binding to lactosylceramide receptor can enhance myeloid progenitor proliferation and neutrophil oxidative burst response, leading to an increase in leukocyte anti-microbial activity. It is also associated with the activation of NF-κB in human neutrophils [59]. Again in other studies, structurally different β-glucans appear to have different affinity toward these receptors. For example, only high molecular weight β-glucans can effectively bind to the lactosylceramide receptor. Therefore, markedly different host responses induced by different β-glucans are expected.

In summary, β-glucans act on a diversity of immune related receptors in particularly Dectin-1 and CR3, and can trigger a wide spectrum of immune responses. The targeted immune cells of β-glucans include macrophages, neutrophils, monocytes, NK cells and dendritic cells (Figure 3). The immunomodulatory functions induced by β-glucans involve both innate and adaptive immune response. β-glucans also enhance opsonic and non-opsonic phagocytosis. Whether β-glucans polarize the T cells subset towards a particular direction remains to be explored.

### Anti-cancer effects of β-glucans

It is becoming clear that β-glucans themselves have no direct cytotoxic effects. Studies implicating the cytotoxic effects of β-glucans were either from studies using crude extracts of β-glucan containing herbs or the use of β-glucan primed monocytes. For β-glucan containing herbs like *Ganoderma lucidum *(Lingzhi), there are other active components such as ganoderic acid from its mycelium [60] and triterpenes from its spore [61-63], which have all been shown to have direct anti-cancer effects independently. We did not find any direct cytotoxic effects of β-glucans on a panel of common cancer cell lines tested including carcinoma, sarcoma, and blastoma. β-glucans also did not trigger any apoptotic pathways and had no direct effect on the telomerase and telomeric length of the cancer cells (unpublished data). In contrast, it stimulated the proliferation of monocytic lineage leukemic cells in-vitro and can facilitate the maturation of dendritic cells derived from leukaemic cells [64]. Hence, whether it is beneficial to apply β-glucans on leukemic patients remains controversial and has to be considered with caution.

In the English literature, there are no clinical trials that directly assessed the anti-cancer effects of purified β-glucans in cancer patients. Most studies were assessing the toxicity profile or underlying immune changes of the cancer patients without addressing on the change of cancer status. In addition, most of the related studies used either crude herbal extracts or a fraction of the extracts instead of purified β-glucans. Therefore, it is difficult to identify whether the actual effects were related to β-glucans or other confounding chemicals found in the mixture.

In a prospective clinical trial of short term immune effects of oral β-glucan in patients with advanced breast cancer, 23 female patients with advanced breast cancer were compared with 16 healthy females control [65]. Oral β-1,3;1,6-glucan was taken daily. Blood samples were recollected on the day 0 and 15. It was found that despite a relatively low initial white cell count, oral β-glucan can stimulate proliferation and activation of peripheral blood monocytes in patients with advanced breast cancer. Whether that can be translated into clinical benefit remains unanswered.

### Clinical trials on anti-cancer effects of natural products with β-glucan

Many edible fungi particularly in the mushroom species yield immunogenic substances with potential anticancer activity [66]. β-glucans are one of the common active components (Table 1). In limited clinical trials on human cancers, most were well tolerated. Among them, lentinan derived from *Lentinus edodes *is a form of β-glucans [67]. Since it has poor enteric absorption, intrapleural, intra-peritoneal [68] or intravenous routes had been adopted in clinical trials which showed some clinical benefit when used as an adjuvant to chemotherapy [69]. Schizophyllan (SPG) or sizofiran is another β-glucan derived from *Schizophyllan commune*. Its triple helical complex β-glucans structure prevents it from adequate oral absorption so an intratumoral route or injection to regional lymph nodes had been adopted [70,71]. In a randomized trial, SPG combined with conventional chemotherapy improved the long term survival rate of patients with ovarian cancer [72]. But whether the prolonged survival can subsequently led to a better cure rate remain unanswered.

**Table 1:** Selected Medicinal Mushroom with β-glucans as Active Components

**Herbs**	**Common Name**	**β-glucans structure**	**Types of β-glucans**
*Lentinus edodes*	Shiitake mushroom	β-1,3;1,6-glucan	Lentinan

*Schizophyllan commune*	Brazilian mushroom, Schizophyllan	β-1,3;1,6-glucan	Schizophyllan (SPG) or sizofiran

*Grifola frondosa*	Maitake mushroom	β-1,3;1,6-glucan with xylose and mannose	Maitake D-Fraction

*Coriolus versicolor*	Yun Zhi	Protein bound β-1,3;1,6-glucan	PSP (polysaccharide peptide) PSK (polysaccharide-Kureha or polysaccharide-K, krestin)

*Ganoderma lucidum*	Lingzhi, Reishi	β-1,3;1,6-glucan	Ganoderma polysaccharides, Ganopoly

*Agaricus blazei*	Brazilian sun-mushroom, Himematsutake mushroom	Protein bound β-1,6-glucan	Agaricus polysaccharides

*Pleurotus ostreatus*	Oyster mushroom, píng gû	β-1,3-glucan with galactose and mannose	Pleuran

*Coprinus comatus*	Shaggy ink cap, lawyer's wig, or shaggy mane	β-1,3-glucan	Coprinus polysaccharides

Maitake D-Fraction extracted from *Grifola frondosa *(Maitake mushroom) was found to decrease the size of the lung, liver and breast tumors in >60% of patients when it was combined with chemotherapy in a 2 arms control study comparing with chemotherapy alone [73]. The effects were less obvious with leukemia, stomach and brain cancer patients [74]. But the validity of the clinical study was subsequently questioned by another independent observer [75]. Two proteoglycans from *Coriolus versicolor *(Yun Zhi) – PSK (Polysaccharide-K) and PSP (Polysaccharopeptide) – are among the most extensively studied β-glucan containing herbs with clinical trials information. However, both PSK and PSP are protein-bound polysaccharides, so their actions are not necessary directly equivalent to pure β-glucans [76]. In a series of trials from Japan and China, PSK and PSP were well tolerated without significant side effects [66,77-81]. They also prolonged the survival of some patients with carcinoma and non-lymphoid leukemia.

*Ganoderma *polysaccharides are β-glucans derived from *Ganoderma lucidum *(Lingzhi, Reishi). While β-glucan is the major component of the Ganoderma mycelium, it is only a minor component in the Ganoderma spore [7]. The main active ingredient in the *Ganoderma *spore extract is triterpenoid which is cytotoxic in nature. In an open-label study on patients with advanced lung cancer, thirty-six patients were treated with 5.4 g/day Ganoderma polysaccharides for 12 weeks with inconclusive variable and results on the cytokines profiles [82]. Another study on 47 patients with advanced colorectal cancer using the same dosage and period again demonstrated similar variable immune response patterns [83]. These results highlight the inconsistency of clinical outcomes in using immune enhancing herbal extracts clinically, which may partly be due to the impurity of the products used.

## Conclusion

The intrinsic differences of the β-glucans derived from different sources will elicit variable immune and anti-cancer responses. We summarized the current limitations of β-glucan research from the literature (Table 2). The limitations are further complicated by the fact that many studies on β-glucan related herbs often used crude extracts rather than purified compounds, therefore the confounding effects of other chemicals cannot be totally ruled out [84]. Careful selection of appropriate β-glucan products with good pre-test quality control is essential if we want to understand and compare the results on how β-glucans act on our immune system and exerting anti-cancer effects. A possibly well-defined β-glucan standard is urgently needed in this field for controlled experiments. So far, there are very few clinical trial data on using purified β-glucans for cancer patients. Future studies should aim to obtain such information so it can assist us in applying β-glucans rationally and effectively to our cancer patients in the future.

**Table 2:** Summary on the Limitations of Current β-glucans Research

**Current Pitfalls or Limitations in β-glucans Research**
• No β-glucan control standard with specific molecular weight and branches are available. Most of the β-glucans publication used zymosan, which is a mixture of chitosan, β-glucans, and cell wall particles.

• Most of the β-glucan containing herbal research are based on extracts rather than purified β-glucans

• No well characterization methods either qualitatively or quantitatively are currently available for assessing and comparing β-glucans from different sources.

• Lack of translational approach to apply knowledge of receptor and signal pathways of β-glucan to animal studies or clinical trials.

• The exact immunological actions and signaling pathway induced by β-glucan are still unclear and have to be further defined.

## Competing interests

The authors declare that there is no conflict of interests, including conflicts of financial nature involving any pharmaceutical or commercial company.

## Authors' contributions

GCFC initiated the concept, wrote and revised the manuscript and creating the illustrations. WKC involved in writing, coordination and revising the manuscript. DMS involved in the preparation and revision of manuscript.
